# Nutritional Risk Factors Associated with Vasomotor Symptoms in Women Aged 40–65 Years

**DOI:** 10.3390/nu14132587

**Published:** 2022-06-22

**Authors:** Alexandra Tijerina, Yamile Barrera, Elizabeth Solis-Pérez, Rogelio Salas, José L. Jasso, Verónica López, Erik Ramírez, Rosario Pastor, Josep A. Tur, Cristina Bouzas

**Affiliations:** 1 Facultad de Salud Pública y Nutrición, Universidad Autónoma de Nuevo León, Monterrey 64460, Mexico; alexandra.tijerinas@uanl.mx (A.T.); yamile.barreracrnz@uanl.edu.mx (Y.B.); elizabeth.solis@uanl.mx (E.S.-P.); rogelio.salasg@uanl.mx (R.S.); jose.jassom@uanl.mx (J.L.J.); veronica.lopezg@uanl.mx (V.L.); erik.ramirezl@uanl.mx (E.R.); 2 Faculty of Health Sciences, Catholic University of Avila, 05005 Avila, Spain; rosario.pastor@ucavila.es (R.P.); cristina.bouzas@uib.es (C.B.); 3 Research Group on Community Nutrition and Oxidative Stress, University of Balearic Islands–IUNICS, 07122 Palma de Mallorca, Spain; 4 Health Institute of the Balearic Islands (IDISBA), 07120 Palma de Mallorca, Spain; 5 CIBER Physiopathology of Obesity and Nutrition (CIBEROBN), Institute of Health Carlos III (ISCIII), 28029 Madrid, Spain

**Keywords:** vasomotor symptoms, risk factors, reproductive aging, menopause, women, Mexico

## Abstract

Vasomotor symptoms (VMS) are the most common symptoms among menopausal women; these include hot flashes and night sweats, and palpitations often occur along with hot flashes. Some studies in Mexico reported that around 50% of women presented with VMS mainly in the menopausal transition. It has been proven that VMS are not only triggered by an estrogen deficiency, but also by nutritional risk factors. Evidence of an association between nutritional risk factors and VMS is limited in Mexican women. The aim of this study is to identify nutritional risk factors associated with VMS in women aged 40–65 years. This is a comparative cross-sectional study, undertaken in a retrospective way. A sample group (*n* = 406 women) was divided into four stages according to STRAW+10 (Stages of Reproductive Aging Workshop): late reproductive, menopausal transition, early postmenopause, and late postmenopause. Hot flashes were present mainly in the early postmenopause stage (38.1%, *p* *≤* 0.001). Two or more VMS were reported in 23.2% of women in the menopausal transition stage and 29.3% in the early postmenopause stage (*p* < 0.001). The presence of VMS was associated with different nutritional risk factors (weight, fasting glucose levels, cardiorespiratory fitness, and tobacco use) in women living in the northeast of Mexico.

## 1. Introduction

Vasomotor symptoms (VMS) are the most common symptoms among menopausal women. These symptoms are short-term menopausal disorders, which include hot flashes and night sweats [1,2], and are often accompanied by palpitations [2]. According to STRAW+10 (Stages of Reproductive Aging Workshop) criteria, which is a “gold standard” that classifies women into stages of reproductive aging, VMS can appear in late menopausal transition but are more common in the early postmenopause [3,4]. Some studies in Mexico reported that VMS in women appear mainly in the menopausal transition, with a prevalence of 45% in women aged 45–55 years [5] and 47.63% in women aged 51–63 years [6].

It has been proven that VMS are not only triggered by estrogen deficiency, but also by different nutritional risk factors that can be modifiable and non-modifiable. Clinical practice guidelines for the menopausal stage [7,8,9], along with several other studies, delimitate that a body mass index higher or equal to 25, hypertension, and tobacco use are nutritional risk factors for VMS [10,11,12]. Their mechanism seems to relate to impaired heat conductance, blood flow, and hormones [12,13]. However, there is limited evidence concerning other factors such as fasting glucose ≥100 mg/dL, poor cardiorespiratory fitness, and excessive total fat intake. 

There is also limited evidence concerning the nutritional factors associated with VMS in Mexican women. In Mexico, the prevalence of VMS has been reported but the associated risk factors have not [5,6]. A previous study has evaluated the social and nutritional factors associated with menopausal symptoms in Mexican women; however, there was no distinction of VMS, as nine different symptoms were arranged into one climacteric group [14]. These studies were carried out in central Mexico and there is limited evidence for the northeast region. The aim of this study is to identify nutritional risk factors associated with VMS in women aged 40–65 years in northeast Mexico.

## 2. Materials and Methods

### 2.1. Design and Subjects

**Figure 1:**
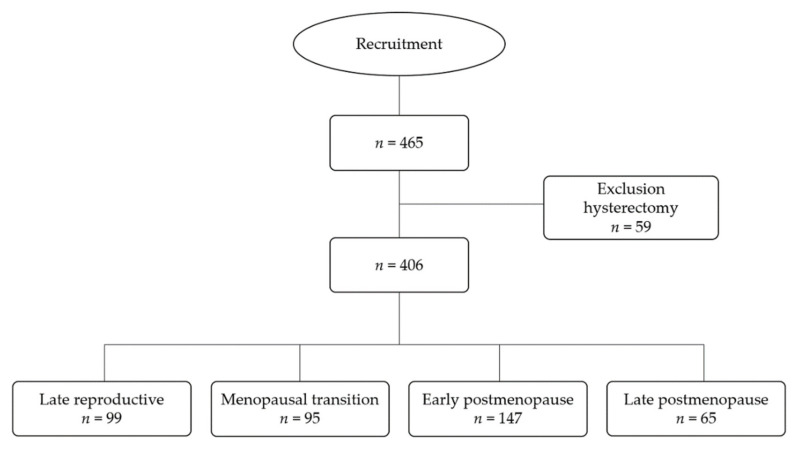
Flowchart of the recruitment of the study population.

This is a cross-sectional study which was carried out from 2015 to 2017. Women enrolled in this study were 40–65 years of age, living in the metropolitan area of Monterrey, in Nuevo León state, Mexico. They were apparently healthy, they voluntarily agreed to participate, and all provided written informed consent. Exclusion criteria included illnesses that affected their habitual eating habits and having undergone a hysterectomy. Women with incomplete data were eliminated from the study analysis (Figure 1). A total of *n* = 406 women were included in the study, representative of the study population (560,115 women aged 40–65 years) of Nuevo León state in the year 2020. This was according to a finite population equation, with 5% error and 95% confidence intervals, considering a VMS proportion of 50% [15].

Women were invited to participate via physical flyers and social media. They were screened for the inclusion criteria via a telephone call and scheduled for an appointment at the Center for Research in Nutrition and Public Health of the Facultad de Salud Pública y Nutrición, Universidad Autónoma de Nuevo León (School of Public Health and Nutrition, Autonomous University of Nuevo León, translated into English).

This study followed the Declaration of Helsinki, and it was approved by the ethics committee of the Facultad de Salud Pública y Nutrición with protocol ID: 15–FaSPyN–SA–11. 

### 2.2. Stages of Reproductive Aging (STRAW+10 Criteria)

A questionnaire was used to obtain data on menstrual cycles, including previous menstrual period, presence of blood flow or amenorrhea, and changes in length between cycles. According to the STRAW+10 criteria [3], women were classified into 4 stages of reproductive aging (Figure 1). Late reproductive was defined as “the presence of blood flow without changes or with short cycles.” Menopausal transition was defined as “the presence of blood flow with long cycles or with at least one interval of amenorrhea ≥60 days,” “amenorrhea <60 days and without changes,” or “amenorrhea ≥60 days.” Early postmenopause was defined as “amenorrhea ≥12 months, but ≤8 years,” and late postmenopause was defined as “amenorrhea >8 years” [3].

### 2.3. Vasomotor Symptoms

Vasomotor symptoms (VMS) were reported in a questionnaire and included hot flashes and night sweats. Palpitations were also registered, as they are often present along with hot flashes [2]. Women had the option to answer yes (presence) or no (absence). After the information was collected, 4 categories were established: (1) absence, (2) presence of hot flashes, (3) presence of night sweats or palpitations, and (4) presence of 2 or more VMS, including hot flashes, night sweats, and palpitations.

### 2.4. Nutrition Assessment

A nutrition assessment of participants was performed according to the Nutrition Care Process of the Academy of Nutrition and Dietetics [16] as follows:

#### 2.4.1. Anthropometric Measurements

Body mass index (BMI) was determined by the formula BMI = weight (kg)/height^2^ (m^2^), using a scale (Seca 874, ± 0.1 kg, Azcapotzalco, Mexico) for the weight and a digital stadiometer (Seca 274, ± 2 mm, Azcapotzalco, Mexico) for the height. BMI was classified as obese ≥30 kg/m^2^, overweight 25–29.9 kg/m^2^, or normal weight 18.5–24.9 kg/m^2^ [17].

#### 2.4.2. Biochemical Data

Venous blood samples were collected at fast, centrifuged at 3500 rpm for 12 min, and serum was obtained. The serum was frozen at −80 °C until assays were performed with the glucose oxidase/peroxidase method. Fasting glucose was obtained using A25 autoanalyzer (software version 4.1.1) (CV = 1.2%) (BioSystems S.A, Barcelona, Spain), according to the Norma Oficial Mexicana NOM–253–SSA1–2012 [9]. Fasting glucose was classified as high ≥100 mg/dL or normal <100 mg/dL [18]. Women on treatments using hypoglycemic drugs were also considered to have high fasting glucose levels.

#### 2.4.3. Nutrition-Focused Physical Exam Findings 

Systolic and diastolic blood pressure measurements were performed to the nearest 1 mmHg using a digital sphygmomanometer, according to the Norma Oficial Mexicana NOM–030–SSA2–2009 [19]. Two readings were taken 5 min apart and the average was calculated. Blood pressure was classified as hypertensive between ≥130 and ≥80 mmHg, elevated between 120–129 and <80 mmHg, or normal between <120 and <80 mmHg [20]. Women on treatments using antihypertensive drugs were considered to be hypertensive.

The cardiorespiratory fitness (CF) of the women was obtained by measuring the walking distance achieved in meters (m) during a six-minute (min) test, in a 15 m × 28 m field. The CF value was reported as meters per minute, using the formula CF = m/6 min. CF was determined as poor at <400 m/6 min or excellent at ≥400 m/6 min [21].

#### 2.4.4. Food and Nutrition-Related History

Total fat intake was assessed by a validated Food Frequency Questionnaire (FFQ) [22]. Women were asked to report the frequency and measurements of their intake of 136 items, including foods and beverages. Total fat and energy intake was analyzed using the software Food Processor^®^ version 15.0 (ESHA Research, Salem, OR, USA) and was reported in grams per day (g/day) [23].

#### 2.4.5. Covariates 

Women also reported tobacco use (yes or no), hormone use (yes or no), and date of birth to determine age (years). Daily physical activity was obtained from the Minnesota leisure-time physical activity (LTPA) questionnaire, in which women reported the frequency and time spent in different activities. Metabolic equivalents per day (MET/d) were calculated [24]. Intake of alcohol in milliliters per day (mL/day), and caffeine in milligrams per day (mg/d), was also analyzed from the FFQ answers (see Section 2.4.4). 

### 2.5. Statistical Analysis

Data were analyzed for normality using the Kolmogorov–Smirnov test. Differences between groups (stages of reproductive aging) were determined using a chi-square test with the Marascuilo procedure to establish the categorical variables. The Kruskal–Wallis test with the Bonferroni adjustment was also used to determine the numerical variables (post hoc test). The presence of VMS in the 4 categories (absence of VMS, presence of hot flashes, presence of night sweats or palpitations, and presence of 2 or more VMS) were reported in frequency and percentage. Differences among categories were determined using a chi-square test with the Marascuilo procedure (post hoc test). 

The dependent variable was the presence of VMS in the 4 categories (yes or no). Independent variables included BMI (obese ≥ 30 kg/m^2^, overweight 25–29.9 kg/m^2^, and normal 18.5–24.9 kg/m^2^), fasting glucose (high ≥100 mg/dL or normal <100 mg/dL), blood pressure (hypertensive between ≥130 and ≥80 mmHg, elevated between 120–129 and <80 mmHg, or normal between <120 and <80 mmHg), cardiorespiratory fitness (poor at <400 m/6 min or excellent at ≥400 m/6 min), total fat intake (excessive >30% from total kcal/d or adequate ≤30% from total kcal/d), and tobacco use (yes or no).

A multivariate logistic regression model was used to define statistical models of the nutritional factors (independent variables) associated with VMS (dependent variable). Odds ratios (OR) with 95% confidence intervals (95% CI) were calculated. Several models were proposed: an unadjusted model (Model 1); an adjusted model (Model 2) including covariates (age (years), hormone use (yes or no), and daily physical activity (MET/day)); and an adjusted model (Model 3) including covariates (stage of reproductive aging (late reproductive, menopausal transition, early postmenopause, or late postmenopause), hormone use (yes or no), daily physical activity (MET/d), alcohol intake (mL/day), and caffeine intake (mg/day)) [25,26,27]. Model 2 was adjusted to include age as a covariate because VMS follows a natural pattern according to lifespan [28]. Model 3 was adjusted to include the stage of reproductive aging because VMS can be present at different stages [4]. 

Prevalence ratios (PR) were calculated using the following formulas, derived from calculated odds ratios (OR) as previously proposed [29,30], where *p*_1_ is the prevalence of VMS in the reference group and z is the coefficient of regression divided by its standard error.

(1) $$\mathrm{PR}=\frac{\mathrm{OR}}{\left(1+p_{1}*[\mathrm{OR}-1]\right)}$$

(2) $$95\%\mathrm{CI}={\mathrm{OR}}^{\left(1\pm (1.96/z)\right)}$$

A *p* value of <0.05 was considered to be statistically significant. All analyses were performed using IBM SPSS^®^ Statistics software, SPSS Inc., Chicago, IL, USA (version 25).

## 3. Results 

Table 1 compares the descriptive characteristics in women grouped by stages of reproductive aging. A total of 24.4% of women were in the late reproductive stage, 23.4% were in menopausal transition, 36.2% were in early postmenopause, and 16.0% were in late postmenopause. The age of menopause was 48.6 years in women at the early postmenopause stage (*n* = 147) and 43.4 years in women in the late postmenopause stage (*n* = 65), while the average menopausal age (*n* = 212) was 47.0 years (data not shown). 

There was a significant difference between stages of reproductive aging in terms of BMI (0.018), fasting glucose (*p* = 0.002), systolic blood pressure (*p* = 0.001), and total fat intake (*p* = 0.039). There were no differences between groups in cardiorespiratory fitness (*p* = 0.877) and tobacco use (*p* = 0.507). Women in the late reproductive stage showed a lower BMI (27.9 kg/m^2^), while women in menopausal transition presented a higher BMI (30.6 kg/m^2^). Fasting glucose was lower in women in the late reproductive stage (95.4 mg/dL) and highest in women in the late postmenopause stage (105.5 mg/dL). Women in the late postmenopause stage showed the highest systolic blood pressure (121.1 mmHg). Total fat intake was higher in women in the late reproductive stage (36.9%), and all groups had intakes above the recommendation of 30% of total energy from fat.

**Figure 2:**
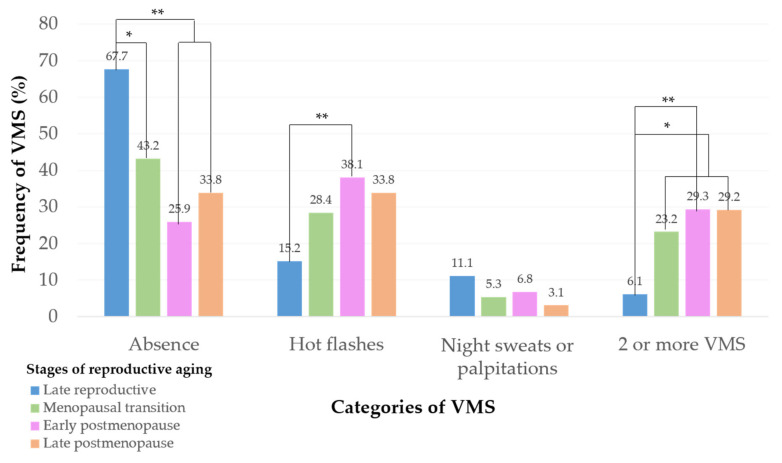
Prevalence of vasomotor symptoms. Data were analyzed using a chi-square test with the Marascuilo procedure (post hoc test). They are expressed as percentages (%). * *p* < 0.01, ** *p* < 0.001.

Prevalence of symptoms are reported as four categories (absence, hot flashes, night sweats or palpitations, and 2 or more VMS) (Figure 2). The study reveals that of all participants (*n* = 406), 41.4% experienced an absence of VMS and 29.6% presented with hot flashes only. An absence of VMS prevailed in women in the late reproductive stage (67.7%). Hot flashes were mainly reported in the early postmenopause stage (38.1%) followed by late postmenopause (33.8%). The presence of either night sweats or palpitations was reported mainly in women in the late reproductive stage (11.1%), although the presence of this category did not differ between different stages (*p* = 0.202). A combination of two or more VMS was reported by 23.2% of women in menopausal transition and 29.3% in early postmenopause; only 6.1% of women in the late reproductive stage presented with two or more VMS.

The association between nutritional risk factors and VMS is shown in Table 2 for unadjusted and adjusted models. In Model 1 (unadjusted model), an overweight BMI (25–29.9 kg/m^2^) denoted a risk for the presence of hot flashes (PR 2.92, 95% CI: 1.66–6.32) (OR 3.24, 95% CI: 1.66–6.33, *p* = 0.001). The presence of either night sweats or palpitations was associated with high levels of fasting glucose (≥100 mg/dL) (OR 2.63, 95% CI: 1.09–6.37, *p* = 0.031) (PR 2.49, 95% CI: 1.09–6.33) and poor cardiorespiratory fitness (OR 15.01, 95% CI: 1.94–115.62, *p* = 0.009) (PR 8.03, 95% CI: 1.95–115.62). In addition, the presence of two or more VMS was associated with high fasting glucose levels (≥100 mg/dL) (OR 2.27, 95% CI: 1.29–3.99, *p* = 0.004) (PR 1.98, 95% CI: 1.29–3.98) and tobacco use (OR 3.19, 95% CI: 1.25–8.11, *p* = 0.015) (PR 2.25, 95% CI: 1.26–8.10). 

After adjusting for age, hormone use, and physical activity level, Model 2 showed very similar results to Model 1 (unadjusted). Excessive weight (BMI 25–29.9 kg/m^2^) was a nutritional risk for the presence of hot flashes (PR 2.93, 95% CI: 1.63–6.51) (OR 3.26, 95% CI: 1.63–6.52, *p* = 0.001). Women were at risk of presenting with either night sweats or palpitations if fasting glucose levels were ≥100 mg/dL (PR 2.38, 95% CI: 1.02–6.11) (OR 2.50, 95% CI: 1.02–6.11, *p* = 0.045) and if they had poor cardiorespiratory fitness levels (PR 7.63, 95% CI: 1.63–112.92) (OR 13.57, 95% CI: 1.63–113.01, *p* = 0.016). Presence of two or more VMS was also associated with high fasting glucose levels (≥100 mg/dL) (PR 1.81, 95% CI: 1.13–3.63) (OR 2.03, 95% CI: 1.13–3.63, *p* = 0.017) and smoking habits (PR 2.12, 95% CI: 1.11–7.55) (OR 2.89, 95% CI: 1.10–7.57, *p* = 0.030). 

Model 3 was adjusted to include the stage of reproductive aging, hormone use, physical activity level, alcohol intake, and caffeine intake (Table 2), denoting consistency in results from Models 1 and 2. The presence of hot flashes was associated with an overweight BMI (25–29.9 kg/m^2^) (PR 2.93, 95% CI: 1.63–6.52) (OR 3.26, 95% CI: 1.63–6.54, *p* = 0.001). Levels of fasting glucose ≥100 mg/dL (PR 2.53, 95% CI: 1.05–6.80) (OR 2.67, 95% CI: 1.04–6.84, *p* = 0.039) and poor cardiorespiratory fitness (PR 8.33, 95% CI: 2.03–129.05) (OR 16.17, 95% CI: 2.02–129.11, *p* = 0.009) are risk factors associated with the presence of night sweats or palpitations. High fasting glucose ≥100 mg/dL (PR 1.75, 95% CI: 1.08–3.48) (OR 1.94, 95% CI: 1.08–3.50, *p* = 0.027) and tobacco use (PR 2.19, 95% CI: 1.15–8.11) (OR 3.05, 95% CI: 1.14–8.13, *p* = 0.025) denoted a risk for the presence of two or more VMS. 

There was no association between blood pressure and total fat intake and the presence of VMS in any proposed model (unadjusted or adjusted).

## 4. Discussion

This observational study in women aged 40–65 years from Nuevo León state, in northeast Mexico, determined the association between nutritional risk factors and the presence of vasomotor symptoms. Menopausal onset was at 47.0 years; this is younger than women living in central Mexico, as previous studies have found that women from Queretaro started menopause at 49.1 years [5] and those from Mexico City at 50.0 years [6]. The onset of menopause at earlier ages, defined as early menopause (<45 years old), [31] could be due to some of the trigger factors that women reported in this study, such as tobacco use and an overweight or obese BMI [32]. Moreover, it has been demonstrated that menopause at early ages is related to a higher risk of cardiovascular disease and mortality, especially in women aged 50–78 years [33]. 

The experience of VMS is similar among Mexican women and follows a pattern that predominates in the early postmenopause stage. In this study, 29.6% of all women showed hot flashes, while 38.1% of women in the early postmenopause stage reported having this specific VMS. In a previous study in central Mexico, 21.3% of women aged 40–60 years reported having hot flashes, mainly during menopausal transition [34]. In other countries, such as India, the United Kingdom, Australia, and the United States of America, hot flashes were more frequently reported during the postmenopausal stage [35,36]. 

The presence of two or more VMS was reported in 22.2% of all women in the current study and was most frequently reported by early postmenopausal women (29.3%). However, previous studies have shown greater incidence of two VMS (hot flashes and night sweats) at the postmenopausal stage; this was found in 50.0% of women aged 24–44 years from the United States of America [11] and in 53.3% of women aged 40–65 years from India [35]. Therefore, in this study, the higher frequency of two or more VMS agrees with the STRAW+10 criteria, which suggests greater a likelihood of symptoms occurring during the early postmenopausal stage [3].

In this study, several women in the late reproductive stage also reported the presence of VMS; 15.2% had hot flashes and 11.1% had either night sweats or palpitations. Previous studies have reported night sweats in 40.0% and hot flashes in 29.0% of late reproductive women aged 35–55 years [37]. It is important to note that VMS can begin at earlier stages, such as the late reproductive stage, although in a low proportion due to estrogen reduction through constant aging and death of follicles [38]. Thus, assessment and diagnosis of VMS is relevant at earlier stages.

The association between nutritional factors and the presence of VMS was analyzed using three regression models to obtain the odds ratio (OR), and a formula using OR values to obtain the prevalence ratio (PR). These demonstrate the risks of women being overweight, having fasting glucose levels above 100 mg/mL, having a cardiorespiratory fitness below 400 m/6 min, and being a smoker. Adjustment of Models 2 and 3 for covariates did not affect the association. Poor cardiorespiratory fitness was the only risk factor with subtle changes in Model 3 that resulted in slightly higher risk and a wider 95% CI. 

Hot flashes were associated with being overweight in this study population (BMI 25–29.9 kg/m^2^) at PR 2.92–2.93 and OR 3.24–3.26 (*p* = 0.001 in all models). Overweight and obese BMIs have previously been associated with the presence of VMS, as reported in the clinical practice guidelines [7,8] and in some studies from Scotland [10], North America [27,39], Australia [40], and South Korea [26]. Women showing a higher BMI tend to have an excess of body fat; visceral fat increases by up to 20% during the postmenopausal stage [41]. In this study, obesity was not associated with hot flashes, which may be because more obese women were at later stages, such as the late postmenopause stage, in which hot flashes were less frequently reported, similar to a previous study [39]. Excessive fat does not allow heat conduction through the skin; therefore, the body tries to release it by maximizing vasodilation, which increases the central body temperature beyond the sweating threshold [13,42]. However, our findings may also suggest that the mechanisms of estrone, associated with a decrease in hot flashes, are naturally occurring in our study of postmenopausal women [39,43].

Fasting glucose levels above 100 mg/dL were associated with either night sweats or palpitations and with two or more VMS. A Swedish study in women aged 50–64 years reported night sweats as the only VMS associated with high glucose levels (*p* < 0.05) [44]. In a longitudinal study of Australian women aged 45–50 years, it was stated that there was a significant association between night sweats and diabetes (OR 1.91, 99% CI: 1.08–3.35, *p* < 0.001) in an adjusted model including similar covariates as this study: age, educational level, length of time, BMI, physical activity level, tobacco use, alcohol intake, menopausal status, and hormone use [45]. Therefore, chronic hyperglycemia could be a strong associated factor. High levels of glucose have been associated with insomnia, because the hypothalamic-pituitary-adrenal axis is altered [46]. This axis also regulates steroid and adrenal secretions [47], so its alteration impacts VMS occurrence.

The association between high blood pressure and VMS could be due to the increased activity of the sympathetic nervous system [48], and thus the increased activity of the adrenalin and sweat glands [49]. Although models in this study showed non-significant results when systolic and diastolic blood pressures were elevated or at hypertension levels, other authors have reported significant associations [44,50]. A study from Sweden in postmenopausal women, aged 50–64 years, reported an association between the presence of night sweats and systolic blood pressure (OR 2.07, *p* < 0.001) [44]. In addition, women in the United States of America aged 45–54 years who were under antihypertensive treatment had 1.80 times greater risk of presenting with hot flashes (*p* <0.05) [50]. 

Fat intake was not associated with the presence of VMS in any model (unadjusted or adjusted). Intake of foods high in fat, especially saturated fat obtained from animal sources such as red or white meat, can increase levels of LDL cholesterol (low-density lipoprotein) [51], and therefore increase the presence of VMS [44]. This is supported by findings of studies in Australian women aged 45–50 years, in which those who followed a high-fat diet presented a significant risk for the presence of both hot flashes and night sweats (unadjusted model, OR 1.16, *p* = 0.002) [52]. Additionally, a study of women aged 40–85 years in the United States of America, who were in menopausal transition and postmenopause, VMS were higher among those who consumed more red and white meat, seafood, and dairy, while an absence of VMS was presented among women consuming a plant-based diet [53]. 

It has been suggested that a decrease in VMS may be observed with improving cardiorespiratory fitness [54,55]. Cardiorespiratory fitness was associated with improved health (*p* < 0.001), emotions (*p* = 0.05), and occupational quality of life (*p* = 0.03), suggesting a positive effect on reduced menopausal symptoms in women aged 45–60 years in the United States of America [55]. However, there has been no reported association with hot flashes and night sweats in Spanish women aged 45–60 years [56]. In the present study, poor cardiorespiratory fitness was the nutritional factor with the highest association to either night sweats or palpitations (PR 7.63–8.33; OR 13.57–16.17, *p* < 0.05). Women who had been physically active since menopausal transition had more protection from VMS. A higher cardiorespiratory fitness level decreases the activation of the sympathetic nervous system, which narrows the blood vessels of the body; in turn, the thermoneutral zone maintains homeostasis [57].

Tobacco use has been reported by different studies as a nutritional risk factor for VMS. Cigarettes are composed of different metals that serve as endocrine disruptors which alter the hormonal balance, so estrogens can be affected until they trigger VMS [12,58]. Women that have been smoking for many years may be at increased risk of having VMS. In a longitudinal study, current smokers aged 24–44 years showed 2.5 times greater risk of having hot flashes and night sweats (95% CI: 1.5–5.3, *p* <0.05) after adjusting for age, hormone level, BMI, hormone use, marital status, and parity [11]. A study of women in the late reproductive stage and the menopausal transition stage from the United States of America, aged 42–52 years, showed an association between the presence of VMS (hot flashes and night sweats), the number of cigarettes smoked (unadjusted model, OR 1.6, 95% CI: 1.3–1.9, *p* < 0.05), and passive smoke exposure (unadjusted model, OR 1.3, 95% CI: 1.2–1.4, 0.05) [59].

### Strengths and Limitations

The main strength of the current study is that it has determined the association between nutritional risk factors and the presence of vasomotor symptoms in women aged 40–65 years from Nuevo León state, in northeast Mexico. The analysis of prevalence and risk is also a strength of this study as it avoids over- or underestimation of this association; however, data could not be compared against other publications. A limitation of this study is the lack of precision in the responses of presence of VMS, as women were questioned in a dichotomous way (yes or no) without considering frequency or intensity. The analysis of some nutritional variables, such as blood pressure and cardiorespiratory function, was limited due to an insufficient number of cases when assessing the categories of VMS. Intakes of subtypes of fat were not analyzed. For future research, it is recommended that women present a similar intake of alcohol and caffeine for more uniformity in those covariates, which may achieve greater precision in adjusted models. In addition, longitudinal studies are needed to infer specific causes or to determine strong risk factors for this specific population.

## 5. Conclusions

The presence of VMS was associated with different nutritional risk factors (weight, fasting glucose levels, cardiorespiratory fitness, and tobacco use) in women living in northeast Mexico. This association was independent of covariates including age, stage of reproductive aging, hormone use, reported physical activity, alcohol intake, and caffeine intake. This evidence supports the need for updating these nutritional risk factors in clinical practice guidelines and its application as an instrument in primary health care services, which assist women at different stages of reproductive aging, at local and national levels.

## Figures and Tables

**Table 1:** Descriptive characteristics of women aged 40–65 years grouped according to *STRAW+10*.

	Late Reproductive	Menopausal Transition	Early Postmenopause	Late Postmenopause	*p*
**Number of women, *n* (%)**	99 (24.4)	95 (23.4)	147 (36.2)	65 (16.0)	
**Age, years**					<0.001
Mean ± SD	44.6 ± 3.3 **^a^**	47.4 ± 3.5 **^b^**	52.6 ± 4.1 **^c^**	56.4 ± 4.0 **^d^**	
Median (Q1, Q3)	44.0 (42.0, 47.0)	48.0 (45.0, 50.0)	52.0 (50.0, 55.0)	53.0 (57.0, 59.0)	
**Drug treatment, *n* (%)**					
*Hormonal*	7 (7.1)	11 (11.6)	13 (8.8)	7 (10.8)	0.715
*Hypoglycemic*	5 (5.1)	8 (8.4)	20 (13.6)	7 (10.8)	0.159
*Antihypertensive*	9 (9.1)	10 (10.5)	21 (14.3)	15 (23.1)	0.057
**BMI, kg/m^2^**					0.018
Mean ± SD	27.9 ± 5.4 **^a^**	30.6 ± 6.7 **^b^**	29.5 ± 5.6 **^ab^**	30.1 ± 5.7 **^ab^**	
Median (Q1, Q3)	27.6 (23.6, 31.1)	29.9 (25.2, 33.9)	28.1 (25.5, 32.8)	29.4 (25.8, 34.5)	
**Fasting glucose, mg/dL**					0.002
Mean ± SD	95.4 ± 32.4 **^a^**	97.5 ± 20.3 **^ab^**	105.1 ± 37.6 **^b^**	105.5 ± 33.3 **^b^**	
Median (Q1, Q3)	91.0 (84.0, 99.0)	95.0 (85.0, 103.0)	98.0 (87.0, 108.0)	98.0 (89.0, 111.5)	
**Blood pressure, mmHg** *Systolic* Mean ± SD Median (Q1, Q3) *Diastolic* Mean ± SD Median (Q1, Q3)	113.3 ± 15.0 **^a^** 110.5 (102.0, 119.5) **^a^** 72.4 ± 11.4 72.0 (65.0, 78.5)	114.9 ± 13.3 **^a^** 114.0 (103.0, 123.5) 73.5 ± 10.0 73.5 (65.5, 82.5)	118.2 ± 17.2 **^ab^** 115.0 (107.0, 126.5) 73.4 ± 12.1 73.0 (66.5, 81.5)	121.1 ± 13.7 **^b^** 122.5 (111.5, 131.5) 72.9 ± 11.0 73.0 (65.0, 80.5)	0.001 0.862
**Cardiorespiratory** **fitness, m/6 min** Mean ± SD Median (Q1, Q3)	549.4 ± 69.0 544.3 (510.1, 601.5)	559.8 ± 70.0 551.9 (513.8, 596.1)	551.3 ± 85. 6 559.0 (507.6, 604.1)	553.3 ± 67.9 541.5 (517.9, 603.4)	0.877
**Total fat intake, %/d**					0.039
Mean ± SD	36.9 ± 6.5 **^a^**	36.6 ± 5.6 **^ab^**	36.2 ± 5.9 **^ab^**	34.3 ± 5.8 **^b^**	
Median (Q1, Q3)	37.1 (33.0, 39.5)	35.9 (32.6, 40.2)	36.1 (32.0, 39.8)	33.4 (30.2, 38.3)	
**Tobacco use, *n* (%)**	6 (6.1)	10 (10.5)	12 (8.2)	8 (12.3)	0.507

BMI: body mass index; SD: standard deviation. Numerical data were analyzed using the Kruskal–Wallis test with the Bonferroni adjustment, and expressed as mean and standard deviation, and median and quartiles (Q1, Q3). Categorical data were analyzed using a chi-square test with the Marascuilo procedure, and expressed as cases and percentages, *n* (%). Superscripts ^a, b, c, d^ denote differences among groups. *p* < 0.05 denotes statistical significance.

**Table 2:** Association between nutritional risk factors and vasomotor symptoms in women aged 40–65 years (*n* = 406).

Vasomotor Symptoms									
	Hot Flashes			Night Sweats or Palpitations			2 or More VMS		
Nutritional Factors	OR (95% CI)	PR (95% CI)	*p*	OR (95% CI)	PR (95% CI)	*p*	OR (95% CI)	PR (95% CI)	*p*
BMI Obese, ≥30 kg/m^2^									
Model 1, unadjusted ^a^	1.32 (0.65–2.67)	1.30 (0.66–2.65)	0.434	1.34 (0.38–4.70)	1.33 (0.39–4.62)	0.643	1.17 (0.57–2.40)	1.16 (0.59–2.31)	0.651
Model 2, adjusted ^b^	1.46 (0.70–3.05)	1.43 (0.71–3.01)	0.306	1.35 (0.38–4.78)	1.34 (0.39–4.65)	0.635	1.27 (0.60–2.68)	1.25 (0.61–2.65)	0.524
Model 3, adjusted ^c^	1.30 (0.62–2.72)	1.28 (0.62–2.73)	0.487	1.24 (0.34–4.49)	1.24 (0.35–4.41)	0.740	1.16 (0.54–2.47)	1.15 (0.55–2.45)	0.697
BMI Overweight, 25–29.9 kg/m^2^									
Model 1, unadjusted ^a^	3.24 (1.66–6.33)	2.92 (1.66–6.32)	0.001	2.49 (0.75–8.26)	2.45 (0.75–8.25)	0.135	1.12 (0.52–2.38)	1.11 (0.53–2.38)	0.768
Model 2, adjusted ^b^	3.26 (1.63–6.52)	2.93 (1.63–6.51)	0.001	2.49 (0.74–8.31)	2.45 (0.75–8.27)	0.136	1.12 (0.52–2.45)	1.11 (0.54–2.31)	0.760
Model 3, adjusted ^c^	3.26 (1.63–6.54)	2.93 (1.63–6.52)	0.001	2.23 (0.65–7.65)	2.20 (0.65–7.61)	0.200	1.08 (0.49–2.39)	1.08 (0.52–2.24)	0.836
BMI Normal 18.5–24.9 kg/m^2^ was reference in all models									
Fasting glucose High, ≥100 mg/dL									
Model 1, unadjusted ^a^	1.47 (0.87–2.51)	1.35 (0.87–2.48)	0.149	2.63 (1.09–6.37)	2.49 (1.09–6.33)	0.031	2.27 (1.29–3.99)	1.98 (1.29–3.98)	0.004
Model 2, adjusted ^b^	1.30 (0.75–2.25)	1.23 (0.75–2.25)	0.347	2.50 (1.02–6.11)	2.38 (1.02–6.11)	0.045	2.03 (1.13–3.63)	1.81 (1.13–3.63)	0.017
Model 3, adjusted ^c^	1.29 (0.74–2.25)	1.23 (0.75–2.23)	0.361	2.67 (1.04–6.84)	2.53 (1.05–6.80)	0.039	1.94 (1.08–3.50)	1.75 (1.08–3.48)	0.027
Fasting glucose Normal <100 mg/dL was reference in all models									
Cardiorespiratory fitness Poor, <400 m/6 min									
Model 1, unadjusted ^a^	1.53 (0.18–12.53)	1.33 (0.19–12.17)	0.689	15.01 (1.94–115.62)	8.03 (1.95–115.62)	0.009	^d^	^d^	^d^
Model 2, adjusted ^b^	1.05 (0.11–9.32)	1.03 (0.14–8.06)	0.963	13.57 (1.63–113.01)	7.63 (1.63–112.92)	0.016	^d^	^d^	^d^
Model 3, adjusted ^c^	1.06 (0.12–8.70)	1.04 (0.14–8.23)	0.956	16.17 (2.02–129.11)	8.33 (2.03–129.05)	0.009	^d^	^d^	^d^
Cardiorespiratory fitness Excellent ≥400 m/6 min was reference in all models									
Blood pressure Hypertensive, systolic ≥130 mmHg or diastolic ≥80 mmHg									
Model 1, unadjusted ^a^	1.47 (0.86–2.50)	1.37 (0.86–2.51)	0.157	1.85 (0.76–4.50)	1.80 (0.77–4.46)	0.170	1.33 (0.73–2.40)	1.29 (0.74–2.39)	0.340
Model 2, adjusted ^b^	1.20 (0.68–2.10)	1.17 (0.69–2.09)	0.519	1.66 (0.67–4.10)	1.63 (0.68–4.06)	0.267	1.11 (0.60–2.07)	1.10 (0.62–2.00)	0.727
Model 3, adjusted ^c^	1.26 (0.72–2.22)	1.21 (0.73–2.17)	0.405	1.86 (0.75–4.63)	1.81 (0.75–4.58)	0.178	1.08 (0.58–2.03)	1.07 (0.52–2.24)	0.792
Blood pressure Elevated, systolic 120–129 mmHg and diastolic 80 mmHg									
Model 1, unadjusted ^a^	0.65 (0.22–1.89)	0.69 (0.22–1.93)	0.437	^d^	^d^	0.979	1.56 (0.60–4.04)	1.47 (0.60–4.03)	0.358
Model 2, adjusted ^b^	0.47 (0.15–1.43)	0.51 (0.15–1.45)	0.189	^d^	^d^	0.986	1.20 (0.44–3.25)	1.18 (0.44–3.24)	0.719
Model 3, adjusted ^c^	0.58 (0.19–1.75)	0.62 (0.19–1.76)	0.336	^d^	^d^	0.989	1.50 (0.54–4.10)	1.43 (0.55–4.10)	0.429
Blood pressure Normal <120 and <80 mmHg was reference in all models									
Total fat intake Excessive >30% of total energy intake									
Model 1, unadjusted ^a^	1.10 (0.60–2.02)	1.09 (0.61–1.97)	0.749	2.50 (0.65–9.49)	2.47 (0.66–9.50)	0.178	1.48 (0.72–3.03)	1.46 (0.72–3.03)	0.283
Model 2, adjusted ^b^	1.41 (0.74–2.69)	1.38 (0.74–2.68)	0.295	2.78 (0.71–10.81)	2.75 (0.72–10.79)	0.140	1.85 (0.87–3.90)	1.80 (0.88–3.90)	0.106
Model 3, adjusted ^c^	1.37 (0.72–2.61)	1.34 (0.73–2.57)	0.328	2.24 (0.57–8.69)	2.22 (0.58–8.67)	0.243	1.97 (0.92–4.21)	1.91 (0.92–4.20)	0.079
Total fat intake Adequate ≤30% of total energy intake was reference in all models									
Tobacco use Yes									
Model 1, unadjusted ^a^	2.14 (0.84–5.42)	1.64 (0.85–5.40)	0.107	2.38 (0.55–10.30)	2.19 (0.55–10.24)	0.244	3.19 (1.25–8.11)	2.25 (1.26–8.10)	0.015
Model 2, adjusted ^b^	1.96 (0.76–5.05)	1.56 (0.76–5.05)	0.163	2.18 (0.49–9.66)	2.03 (0.50–9.60)	0.303	2.89 (1.10–7.57)	2.12 (1.11–7.55)	0.030
Model 3, adjusted ^c^	2.04 (0.78–5.31)	1.60 (0.79–5.26)	0.140	2.31 (0.52–10.29)	2.14 (0.52–10.22)	0.270	3.05 (1.14–8.13)	2.19 (1.15–8.11)	0.025
Tobacco use *No* was reference in all models									

VMS: vasomotor symptoms. BMI: body mass index. OR: odds ratio. PR: prevalence ratio. 95%CI: 95% confidence interval. ^a^ Model 1: unadjusted. ^b^ Model 2: adjusted for age, hormone use, and physical activity level. ^c^ Model 3: adjusted for stage of reproductive aging, hormone use, physical activity level, alcohol intake, and caffeine intake. ^d^ Insufficient cases for a statistical result. *p* < 0.05 denotes statistical significance.

## Data Availability

There are restrictions on the availability of data for this trial, due to the signed consent agreements around data sharing, which only allow access to external researchers for studies following the project purposes. Those wishing to access the trial data used in this study can make a request to pep.tur@uib.es.
